# Association Between Unclean Cooking Fuel Use and Hearing Problems Among Adults Aged ≥ 65 Years, a Cross‐Sectional Study

**DOI:** 10.1002/hsr2.70717

**Published:** 2025-04-21

**Authors:** Robert C. MacKinnon, Lee Smith, Guillermo F. López Sánchez, Damiano Pizzol, Peter Allen, Saima Rajasingam, Shahina Pardhan, Pinar Soysal, Nicola Veronese, Laurie Butler, Yvonne Barnett, Hans Oh, Jae Il Shin, Ai Koyanagi

**Affiliations:** ^1^ Vision and Hearing Sciences Research Centre Anglia Ruskin University Cambridge UK; ^2^ Centre for Health Performance and Wellbeing Anglia Ruskin University Cambridge UK; ^3^ Department of Public Health Sciences, Division of Preventive Medicine and Public Health, School of Medicine University of Murcia Murcia Spain; ^4^ Italian Agency for Development Cooperation Khartoum Sudan; ^5^ Vision and Eye Research Institute, School of Medicine, Faculty of Health, Education, Medicine and Social Care Anglia Ruskin University Cambridge UK; ^6^ Department of Geriatric Medicine, Faculty of Medicine Bezmialem Vakif University Istanbul Turkey; ^7^ Faculty of Medicine Saint Camillus International University of Health Sciences Rome Italy; ^8^ Suzanne Dworak Peck School of Social Work University of Southern California Los Angeles California USA; ^9^ Department of Pediatrics Yonsei University College of Medicine Seoul Republic of Korea; ^10^ Severance Underwood Meta‐Research Center, Institute of Convergence Science Yonsei University Seoul Republic of Korea; ^11^ Research and Development Unit Parc Sanitari Sant Joan de Déu Barcelona Spain

**Keywords:** epidemiology, hearing problems, low‐ and middle‐income countries, older adults, unclean cooking fuel

## Abstract

**Background and Aims:**

Literature suggests that outdoor air pollutant exposure is associated with hearing problems, but examination of this link has not extended to any potential association between hearing ability and the use of unclean cooking fuels. The current paper investigates whether such a link exists, utilizing a large sample of older adults from low‐ and middle‐income countries (LMICs) where such fuels are commonly used.

**Methods:**

Data from the Study on global AGEing and adult health (SAGE) were analyzed. This is a nationally representative and cross‐sectional data set collected for the World Health Organization for residents of South Africa, China, Ghana, India, Mexico, and Russia. A range of “unclean” cooking fuels were assessed, namely agriculture or crop, animal dung, coal or charcoal, Kerosene or paraffin, shrubs or grass, and wood. Hearing problems referred to the interviewer‐rated presence of this condition. Statistical analysis was done using multivariable logistic regression.

**Results:**

The present work analyzed data from 14,585 individuals aged ≥ 65 years [mean (SD) age 72.6 (11.5) years; 55.0% females]. In the overall sample and in the final adjusted model, unclean cooking fuel use was associated with a significantly increased risk of hearing problems (OR = 1.68 (95% CI = 1.22–2.30). This association was significant for females (OR = 2.36; 95% CI = 1.53–3.63) but not for males (OR = 1.20; 95% CI = 0.79–1.81).

**Conclusion:**

Unclean cooking fuel use is associated with an increased risk of hearing problems among adult residents of LMICs over 65 years of age, particularly among females. Findings from this study support the development of Sustainable Development Goal 7 (United Nations), which advocates for fairer and more sustainable access to modern energy, as well as a means to prevent avoidable hearing problems.

## 1 Introduction

Hearing impairment may be defined as not being able to hear as well as someone with normal hearing, taken as having hearing thresholds of 20 decibels hearing level (dB HL) or better in both ears across the clinically tested frequency range of human hearing (250 Hz–8 kHz) [1]. It can be of various severity and can affect one or both ears, with a disabling hearing impairment described as having an average hearing threshold of ≥ 35 dB HL across these frequencies [2]. Approximately 1.57 billion people worldwide have impaired hearing, which impacts communication and everyday life [3]. The risk of hearing impairment increases with increasing age, rising to more than one in four aged over 60 years being affected by significant hearing impairment [2]. Importantly, around 80% of those with a disabling loss reside in low‐ and middle‐income countries (LMICs). Such a striking prevalence of hearing impairment is of concern as multiple poor health outcomes and comorbidities are associated with uncorrected hearing impairment. For example, in an umbrella review on hearing impairment and all health outcomes, hearing impairment was associated with an increased risk of cognitive impairment as well as a low quality of life [4]. Moreover, hearing impairment has significant economic impacts that extend beyond health and education, with the main costs resulting from loss of employment and poor quality of life [5]. For example, a conservative estimate of the overall global annual cost of hearing impairment is approximately 980 billion dollars [2], and 57% of these costs are borne by LMICs (rising to 83% when using mean world GDP per capita) owing to limited availability of audiologists, and costs of screening and treatment. Therefore, interventions that aid in the prevention of hearing impairment in LMICs are needed. To inform targeted interventions, risk factors of hearing impairment should be identified. While several risk factors for hearing problems have been reported (e.g., aging, loud noises, and some diseases), one risk factor that could be of interest for hearing impairment is the use of unclean fuels for cooking, which is especially prevalent in LMICs.

Specifically, unclean cooking fuels are those not meeting the WHO definitions recommended in their global air quality guidelines [6]. This definition includes liquid fuels such as kerosene and paraffin, and solid fuels such as charcoal, coal, agricultural and crop residues, wood, animal dung, and shrubbery or grass. It has been estimated in 2000, approximately 3.08 billion people used unclean cooking fuel as their primary or only cooking fuel [7]. Out of these 3 billion people, almost 87% reside in LMICs [8]. More recent estimates suggest that the global total unclean cooking fuel use has fallen to 2.1 billion people in 2022 [9, 10], representing a positive trend but highlighting the large proportion of the global population the issue still affects. Unclean cooking fuel may increase the risk for hearing impairment or problems via pollutants (e.g., particulate matter) from unclean cooking fuels that can increase oxidative stress, which may lead to the loss of hair cells or synapses in the ear, permanent neurosensory damage, and concomitant hearing loss [11]. At least one study in an adult Korean population (*n* = 15,000) found that long‐term exposure to airborne pollutants (NO_2_, CO, and PM_10_) was associated with mid (speech)—and high‐frequency hearing impairment [6]. Additionally, long‐term exposure to SO_2_ was associated with mid‐frequency hearing impairment in an adult population [11]. However, to date, no specific investigation has been identified on the use of unclean cooking fuels and hearing impairment or problems.

Thus, with the very high prevalence of hearing problems, especially among older people residing in LMICs, and the plausible mechanism for the use of unclean cooking fuels to cause hearing impairment or problems, we hypothesize that using unclean cooking fuel will be associated with an increased risk of hearing problems. The present study aims to investigate the association between unclean cooking fuel use and hearing problems in a large sample of ~15,000 adults residing in LMICs aged ≥ 65 years.

## 2 Methods

### 2.1 Data Source

The data source for the study is Wave 1 of the WHO Study, the Study on Global Ageing and Adult Health (SAGE), a longitudina study with nationally representative samples of people aged ≥ 50 years in six LMICs [12]. The methodology of this large‐scale survey is documented and separately published by its authors [7]. The survey, conducted between 2007 and 2010, investigated the populations of South Africa, Russia, Mexico, India, Ghana, and China, all of which were classified at the time of the study as LMICs. Multistage clustered sampling was used to capture representative samples for each nation. In each case, adults ≥ 18 years were recruited, with participants ≥ 50 years being over‐sampled. Data were collected by face‐to‐face interviews using trained interviewers utilizing a standardized questionnaire, which was translated for each nation. Response rates ranged from 93% (China) to 53% (Mexico), with intermediate rates from Russia (83%), Ghana (81%), South Africa (75%), and India (68%). Sampling weights were adjusted for nonresponse and the structure of each population according to guidance from the Statistical Division of the United Nations. The study was granted ethical approval by the Review Committee of the WHO alongside regional ethical approval by local committees. Ethical approval was obtained from the WHO Ethical Review Committee and local ethics research review boards.

### 2.2 Hearing Problems

At the end of the survey, the interviewer provided information on whether they perceived each participant to have a hearing problem during the survey, with binary “yes” and “no” response options only. The study utilizes a population aged 50 years and older, which makes it suitable for the detection of hearing loss that may develop over time but is not yet attributable to ageing.

### 2.3 Cooking Fuel

SAGE asked its respondents, “What type of fuel does your household mainly use for cooking?” to establish which primary fuel was being used. Response options were shrubs/grass, animal dung, agriculture/crop, wood, coal/charcoal (solid fuels), kerosene/paraffin, electricity, and gas. As in previous SAGE reporting, these were transformed into the binary responses of “clean fuels” (electricity and gas) and “non‐clean fuels” (remaining fuels). The location of fuel usage was assessed with the question “Where is cooking usually done?” with responses coded as “in a room used for sleeping” or “else” (i.e., outdoors or else in a separate building or room).

Respondents who selected solid fuels as categorized above were asked which type of stove was used to burn them (“In this household, is food cooked on an open fire, an open or closed stove?”) with similar dichotomization of responses as “closed stove” or “open fire/stove” [13]. They were also asked, “Does the fire/stove have a chimney, hood, or neither?” with responses collapsed to “neither” or “chimney or hood” [13].

### 2.4 Control Variables

Control variables were identified with reference to prior articles [11]. These included country, sex, age, level of education (tertiary, secondary, primary, or less) and income (quintiles of wealth), unemployment (not engaged in paid work for 2 or more days of the last 7 days), residential setting (rural or urban), smoking (past, current, never), diabetic status (self‐reported lifetime diagnosis), hypertensive status (one or more of self‐report, diastolic blood pressure ≥ 90 mmHg, or systolic ≥ 140 mmHg), and body mass index (BMI). Widely accepted BMI categorizations were used for “obese” (over 30.0 kg/m^2^), “overweight” (25.0–29.9 kg/m^2^), “normal” (18.5–24.9 kg/m^2^), and “underweight” (less than 18.5 kg/m^2^) [14]. The Asian cut‐offs of overweight (BMI 23.0–27.5 kg/m^2^) and obese (BMI ≥ 27.5 kg/m^2^) were also used for sensitivity analysis [15].

### 2.5 Statistical Analysis

Statistical analyses were conducted with Stata 14.2 (Stata Statistical Software: Release 14. College Station, TX: StataCorp LLC.). An analysis strategy similar to that previously used to investigate other health sequelae was employed [16, 17]. Only data from respondents 65 years and over was analyzed due to the increased risk of hearing loss in this group (e.g., Royal National Institute for Deaf People RNID [18]). A complete case analysis was performed for all analyses. The difference in characteristics as grouped by hearing problems was tested using Chi‐squared tests, with the exception of age (Student's *t*‐test). Multivariable logistic regression analysis was done to assess the association between unclean cooking fuel use (“exposure”) and hearing problems (“outcome”). To check for an interaction effect of sex on any relation between exposure and outcome, the product term “unclean cooking fuel use X sex” was introduced to the model. Since preliminary analysis identified that there was indeed an interaction effect, the analysis was then stratified by sex. Furthermore, analysis with individual fuel type (e.g., agricultural, coal) and ventilation type as the “exposure” was also performed. The analysis of ventilation type was necessarily restricted to those using solid fuels as these data only existed for this subgroup. All regression analyses included the full set of control variables noted above (i.e., age, sex, wealth, etc.). As in previously reported SAGE analyses, the inclusion of dummy variables for each country in the model allowed for the country to be adjusted for [19, 20]. For all analyses, the complex design of the study and sample weightings were accounted for. Regression analysis results are presented as odds ratios (ORs), associated confidence intervals (95% CIs), with statistical significance set at *p* < 0.05.

## 3 Results

**Figure 1:**
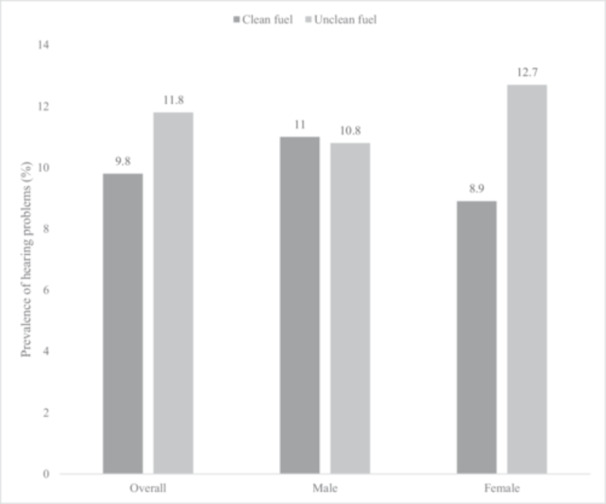
Prevalence of hearing problems by type of cooking fuel (overall and by sex).

The present work analyzed data from 14585 individuals aged ≥ 65 years. Table 1 presents the sample characteristics. The mean age of the sample was 72.6 (SD = 11.5) years and 55.0% were females. The sample sizes from each country were: China, *n* = 5360; India, *n* = 2441; Ghana, *n* = 1975; Russia, *n* = 1950; South Africa, *n* = 1484; Mexico, *n* = 1375. The prevalence of hearing problems and unclean cooking fuel use were 10.7% and 45.9%, respectively. Lower levels of wealth and education, unemployment, rural settings, and hypertension were significantly more common among those with hearing problems, while people with hearing problems were also older. The level of hearing problems was higher among unclean cooking fuel users in the overall sample and females (Figure 1). For example, in females, the prevalence of hearing problems among those who use clean cooking fuel was 8.9%, but this increased to 12.7% among those who use unclean cooking fuel. After adjustment for potential confounders, in the overall sample, unclean cooking fuel use was associated with a significant 1.68 (95% CI = 1.22–2.30) times higher odds for hearing problems (Table 2). This association was significant in females only (OR = 2.36; 95% CI = 1.53–3.63; males OR = 1.20; 95% CI = 0.79–1.81). Hearing problems were not significantly associated with cooking ventilation status (Table 3). Finally, in comparison to using clean cooking fuel, the use of coal/charcoal, agriculture/crop, and shrubs/grass were all associated with significantly higher odds for hearing problems (Table 4). The results remained almost the same when the Asian cut‐off for BMI was used for adjustment.

**Table 1:** Sample characteristics (overall and by hearing problems).

			Hearing problems		
Characteristic		Overall	No	Yes	*p* value^a^
Age (years)	Mean (SD)	72.6 (11.5)	72.1 (10.9)	75.8 (13.4)	< 0.001
Sex	Female	55.0	55.0	54.2	0.738
	Male	45.0	45.0	45.8	
Wealth	Poorest	21.7	21.0	27.7	0.003
	Poorer	21.0	21.0	20.8	
	Middle	20.4	20.2	21.5	
	Richer	17.5	18.0	12.9	
	Richest	19.4	19.8	17.1	
Education	≤ Primary	63.7	62.6	70.3	0.010
	Secondary	29.9	30.7	24.7	
	Tertiary	6.4	6.7	5.0	
Unemployed	No	21.6	22.7	13.2	< 0.001
	Yes	78.4	77.3	86.8	
Setting	Urban	50.6	51.3	44.6	0.020
	Rural	49.4	48.7	55.4	
Smoking	Never	62.2	62.5	59.7	0.045
	Current	29.3	29.4	29.0	
	Past	8.5	8.1	11.3	
Body mass index (kg/m^2^)	< 18.5	19.3	19.3	21.0	0.081
	18.5–24.9	46.4	45.9	50.4	
	25.0–29.9	23.9	24.3	19.2	
	≥ 30	10.4	10.5	9.3	
Diabetes	No	91.4	91.3	92.5	0.308
	Yes	8.6	8.7	7.5	
Hypertension	No	36.6	37.4	31.9	0.020
	Yes	63.4	62.6	68.1	

*Note:* Data are % unless otherwise stated.

Abbreviation: SD Standard deviation.

^a^ *p* value was obtained by Chi‐squared tests for categorial variables, and Student's *t*‐tests for continuous variables.

**Table 2:** Association between unclean cooking fuel use (or covariates) and hearing problems (outcome) estimated by multivariable logistic regression (overall and by sex).

	Overall			Male			Female		
	OR	95% CI	*p* value	OR	95% CI	*p* value	OR	95% CI	*p* value
Unclean cooking fuel use									
No	1.00			1.00			1.00		
Yes	1.68	[1.22, 2.30]	0.001	1.20	[0.79, 1.81]	0.392	2.36	[1.53, 3.63]	< 0.001
Age (years)	1.08	[1.07, 1.10]	< 0.001	1.08	[1.06, 1.11]	< 0.001	1.08	[1.06, 1.11]	< 0.001
Sex									
Female	1.00								
Male	1.15	[0.90, 1.46]	0.266						
Wealth									
Poorest	1.00			1.00			1.00		
Poorer	0.84	[0.63, 1.11]	0.224	0.75	[0.51, 1.11]	0.148	0.95	[0.63, 1.42]	0.795
Middle	0.97	[0.71, 1.34]	0.871	0.93	[0.60, 1.42]	0.721	1.02	[0.65, 1.59]	0.942
Richer	0.72	[0.51, 1.01]	0.061	0.55	[0.34, 0.90]	0.016	0.91	[0.56, 1.48]	0.708
Richest	1.00	[0.66, 1.51]	0.983	0.70	[0.46, 1.07]	0.1	1.34	[0.71, 2.53]	0.361
Education									
≤ Primary	1.00			1.00			1.00		
Secondary	0.76	[0.53, 1.10]	0.146	0.83	[0.52, 1.32]	0.434	0.66	[0.41, 1.07]	0.093
Tertiary	0.68	[0.40, 1.17]	0.166	0.98	[0.55, 1.75]	0.952	0.21	[0.08, 0.57]	0.002
Unemployed									
No	1.00			1.00			1.00		
Yes	1.80	[1.39, 2.33]	< 0.001	1.23	[0.88, 1.72]	0.232	3.11	[1.97, 4.92]	< 0.001
Setting									
Urban	1.00			1.00			1.00		
Rural	1.08	[0.80, 1.46]	0.611	1.18	[0.79, 1.77]	0.427	1.02	[0.70, 1.50]	0.918
Smoking									
Never	1.00			1.00			1.00		
Current	1.05	[0.81, 1.37]	0.707	0.97	[0.70, 1.35]	0.87	1.16	[0.77, 1.74]	0.472
Past	1.36	[0.96, 1.94]	0.083	1.19	[0.78, 1.81]	0.411	1.70	[0.86, 3.37]	0.126
Body mass index (kg/m^2^)									
< 18.5	1.02	[0.75, 1.39]	0.901	0.74	[0.48, 1.15]	0.175	1.27	[0.84, 1.92]	0.265
18.5–24.9	1.00			1.00			1.00		
25.0–29.9	0.69	[0.54, 0.88]	0.003	0.90	[0.60, 1.36]	0.629	0.52	[0.36, 0.77]	0.001
≥ 30	0.75	[0.50, 1.12]	0.154	1.15	[0.57, 2.31]	0.697	0.55	[0.36, 0.84]	0.006
Diabetes									
No	1.00			1.00			1.00		
Yes	1.07	[0.76, 1.50]	0.691	1.00	[0.57, 1.76]	0.999	1.20	[0.79, 1.83]	0.398
Hypertension									
No	1.00			1.00			1.00		
Yes	1.18	[0.95, 1.48]	0.133	1.38	[1.00, 1.89]	0.049	1.05	[0.79, 1.39]	0.741

*Note:* Model is adjusted for all variables in the Table and country.

Abbreviations: CI, confidence interval; OR, odds ratio.

**Table 3:** Association between cooking ventilation and hearing problems (outcome) estimated by multivariable logistic regression.

Cooking ventilation	OR	95% CI	*p* value
Stove			
Closed stove	1.00		
Open stove or fire	0.85	[0.63, 1.16]	0.303
Chimney/hood			
Chimney or hood	1.00		
Without chimney or hood	1.20	[0.79, 1.82]	0.404
Cooking place			
In a separate room/building used as kitchen or outdoor	1.00		
In a room used for living or sleeping	0.96	[0.57, 1.61]	0.864

*Note:* Models are adjusted for age, sex, wealth, education, unemployment, setting, smoking, body mass index, diabetes, hypertension, and country. The sample is restricted to those using solid fuels (coal or biomass fuels).

Abbreviations: CI, confidence interval; OR, odds ratio.

**Table 4:** Association between different types of unclean cooking fuels and hearing problems (outcome) estimated by multivariable logistic regression.

	OR	95% CI	*p* value
Clean^a^	1.00		
Kerosene/paraffin	0.61	[0.25, 1.44]	0.258
Coal/charcoal	1.73	[1.19, 2.51]	0.004
Wood	1.31	[0.88, 1.95]	0.186
Agriculture/crop	2.34	[1.38, 3.95]	0.002
Animal dung	1.65	[0.65, 4.20]	0.295
Shrubs/grass	2.40	[1.58, 3.66]	< 0.001

*Note:* Models are adjusted for age, sex, wealth, education, unemployment, setting, smoking, body mass index, diabetes, hypertension, and country.

Abbreviations: CI, confidence interval; OR, odds ratio.

^a^ Clean cooking fuel referred to gas and electricity.

## 4 Discussion

### 4.1 Main Findings

In the present representative sample of older adults from multiple LMICs (*n* = 6), unclean cooking fuel use was significantly associated with a 1.68 (95% CI = 1.22–2.30) times higher odds for hearing problems in the overall sample. Interaction analysis showed that there is significant effect modification by sex in this association, with the strength of the association being particularly pronounced among females (OR = 2.36; 95% CI = 1.53–3.63). In addition, we found that cooking ventilation type is not associated with hearing problems, while in terms of individual cooking fuel types, coal/charcoal, agriculture/crop, and shrubs/grass were significantly associated with higher odds for hearing problems. To the best of our knowledge, we report for the first time that unclean cooking fuel use is associated with hearing problems.

### 4.2 Interpretation of Findings

While there are no prior studies on unclean cooking fuel use and hearing problems, our study results are in line with a study on “air pollutants” and hearing problems conducted in Korea [11]. There are plausible pathways that may explain the observed association between unclean cooking fuel use and hearing problems. Considering traditional Bradford‐Hill Criteria [21], several conditions are met or partially met (e.g., strength, plausibility, coherence), which strengthens the case for an association and its further investigation. The cochlea have high metabolic demands of hair cells in response to sound stimulation and, thus, are vulnerable to oxidative stress [22] that may lead to hair cell apoptosis in the ear and permanent cochlear degeneration [11]. Indeed, cooking with biomass fuels has been shown to exacerbate oxidative stress [23].

An alternative explanation for this increase in hearing difficulties is linked to the potential presence of acrolein in the cooking environment. Acrolein is produced in the burning of wood, fossil fuels, and tobacco and is a known neurotoxin and ototoxin. It has been observed to induce mitochondrial dysfunction and endoplasmic reticulum stress in cochlear nucleus neurons, contributing to their injury and programmed cell death [24]. Damage to the cochlear nucleus can result in hearing impairment, in particular, auditory neuropathy spectrum disorder, characterized by difficulties in speech discrimination or sound localization that are often worse than those predicted from the pure tone audiometric thresholds [25]. The involvement of acrolein is highly consistent with this study's findings that biomass fuels (coal/charcoal, agriculture/crop, and shrubs/grass) were associated with increased odds of hearing difficulties.

Furthermore, there is substantial evidence that pollutants resulting from incomplete combustion of biomass fuels in the domestic environment – in particular, suspended particulate matter and nitrogen oxide—increase the risk of upper respiratory tract infections, which can result in chronic otitis media (infection of the middle ear). Otitis media is typically characterized by fluid in the middle ear cavity and conductive hearing loss. If otitis media persists, this can lead to mastoiditis (infection of the mastoid) and, in turn, to permanent deafness [26]. It is notable that chronic otitis media is particularly common in LMICs (although the disease burden is difficult to estimate accurately with current evidence) [27].

Interestingly, in our study, there was significant effect modification by sex in the association between unclean cooking fuel use and hearing problems, where the association was more pronounced among females. This association may be explained by higher levels of exposure to pollutants from using unclean cooking fuels among females in LMICs. Indeed, in such settings, females tend to take the majority of the burden of household chores, including cooking, and thus experience greater exposure to both pollutants [28, 29].

### 4.3 Implication of Study Findings

Findings from the present study support the importance of Sustainable Development Goal 7 described by the United Nations related to sustainable and fair access to clean energy [30]. The present study adds, for the first time, hearing problems to the list of associated negative outcomes that can be avoided by transitioning away from unclean cooking fuel usage. When taken together with the previously demonstrated associations of their negative impact on health, which spans mental, cardiovascular, and respiratory domains [21, 22, 23], the case is more compelling than ever to prioritize the cessation of their use and empower LMIC communities to do so.

### 4.4 Strengths and Limitations

The SAGE study from which these data for analysis are derived has strengths in terms of the large study cohort recruited and investigated. It focused on a highly relevant and under‐investigated population in the form of LMIC residents and older residents of these countries most at risk of hearing loss. Limitations are present, though, which should be acknowledged in spite of a statistically significant effect being identified. First, hearing problems were only based on observation of the interviewer and did not consist of audiological assessment (e.g., through ear examination and objective or subjective audiological tests), which can provide more accurate information on the extent and underlying cause of reported hearing problems. While observational and subjective assessments have been argued to be important in assessing the activity and participation dimensions of disease [1]. Additionally, there was no exploration of additional hearing symptoms such as tinnitus or dizziness, which would be suitable areas of expansion for further work. Second, the study was cross‐sectional in nature, and thus, the direction of the association is not known. However, it is highly unlikely that hearing problems drive unclean cooking fuel use. Third, the level and length of exposure to unclean cooking fuel use were not measured. Future studies should consider including this variable as longer exposure to unclean cooking fuel can theoretically lead to a higher risk of hearing problems. Finally, we lacked information on heat exposure, which can be induced by unclean cooking fuel use and can be a risk factor for hearing loss [31]. Thus, residual confounding may exist due to this factor. These factors could be addressed in subsequent surveys to help improve the generalizability and strength of our findings.

## 5 Conclusion

In this large representative sample of older adults residing in LMICs, unclean cooking fuel use was associated with higher odds of hearing problems, especially among females. The findings of our study support the implementation of Sustainable Development Goal 7 also for the prevention of hearing problems, but future studies from diverse settings are necessary to assess the extent to which our findings can be generalized to other settings and, indeed, other measures of hearing health.

## Author Contributions

**Robert C. MacKinnon:** writing – original draft. **Lee Smith:** writing – original draft. **Guillermo F. López Sánchez:** writing – review and editing. **Damiano Pizzol:** writing – review and editing. **Peter Allen:** writing – review and editing. **Saima Rajasingam:** writing – review and editing. **Shahina Pardhan:** writing – review and editing. **Pinar Soysal:** writing – review and editing. **Nicola Veronese:** writing – review and editing. **Laurie Butler:** writing – review and editing. **Yvonne Barnett:** writing – review and editing. **Hans Oh:** writing – review and editing. **Jae Il Shin:** writing – review and editing. **Ai Koyanagi:** writing – original draft.

## Conflicts of Interest

The authors declare no conflicts of interest.

## Transparency Statement

The lead authors, Guillermo F. López Sánchez and Jae Il Shin, affirm that this manuscript is an honest, accurate, and transparent account of the study being reported; that no important aspects of the study have been omitted; and that any discrepancies from the study as planned (and, if relevant, registered) have been explained.

## Data Availability

The data that support the findings of this study are available from the corresponding author upon reasonable request.
